# Intra-abdominal aggressive fibromatosis: a single-center case series of 12 patients focusing on surgical management and clinicopathological features

**DOI:** 10.1186/s12876-026-04867-6

**Published:** 2026-04-24

**Authors:** Yuxin Teng, Fuyuan Liu, Zhuohui Ren, Niu Dai, Zhengcai Liu, Shuqiang Yue

**Affiliations:** Department of General Surgery, Xijing Hospital, Air Force Medical University, 127 West Changle Street, Xi’an, Shaanxi 710032 China

**Keywords:** aggressive fibromatosis, clinical characteristic, surgery, treatment

## Abstract

**Background:**

Intra-abdominal aggressive fibromatosis (AF) is a rare disease with invasive growth and local recurrence. However, few studies have reported the clinical and pathological features of intra-abdominal AF. The aim of this study is to systematically elucidate the clinical characteristics, imaging manifestations, treatment and pathological features of intra-abdominal AF.

**Methods:**

A total of 12 patients with intra-abdominal AF diagnosed in Xijing Hospital, First Affiliated Hospital of Air Force Medical University from April 2021 to September 2024 were included. All the specimens underwent routine staining and immunohistochemical analysis. The study was approved by the Ethics Review Committee of Xijing hospital.

**Results:**

A total of 12 patients with intra-abdominal AF were enrolled (median age: 33 years; range: 16–59 years). Tumors were located in the retroperitoneum (*n* = 8) or small intestinal mesentery (*n* = 4), with a mean maximum diameter of 9 cm (6–30 cm). Computed tomography typically showed irregular soft tissue masses with heterogeneous density and enhancement. All patients underwent R0 resection; notably, 10 (83.3%) required combined organ resection due to involvement of adjacent organs or major vessels. Postoperatively, four patients (33.3%) developed mild, transient complications, all of which resolved with conservative management during the same hospitalization. No adjuvant therapy was administered. Immunohistochemistry revealed nuclear β-catenin positivity in all 12 cases (100%), SMA positivity in seven (58.3%), Vimentin positivity in four (33.3%), and a Ki-67 index of 2–20%. CD117 was negative in all cases. At a median follow-up of 23 months (7–48 months), no recurrence or metastasis was observed.

**Conclusion:**

Intra-abdominal AF is a rare disease with a high reported local recurrence rate. Early surgery can achieve complete removal in experienced medical centers, and complication rates are acceptable, with good short-term prognosis.

## Introduction

Aggressive fibromatosis (AF), which is also known as desmoid-type fibromatosis or desmoid tumor, is a disease of monoclonal fibroblast proliferation occurring in deep tissue. It is typically characterized by aggressive growth with a tendency to local recurrence but without distant metastasis [1]. AF is a very rare disease, occurring in approximately 5–6 cases per one million people per year, accounting for only 0.03% of all diagnosed tumors [2]. It mainly occurs between 15 and 60 years of age, with a peak age of 30–40 years, and the incidence rate is higher in women than in men [3].

AF can occur in any part of the body, including the extremities, trunk, head and neck, and abdomen. According to the site of occurrence, it can be classified into three types: extra-abdominal AF, abdominal wall AF, and intra-abdominal AF [4]. Among them, intra-abdominal AF has a lower incidence rate, is prone to misdiagnosis, exhibits poor prognosis, and poses numerous challenges in clinical management. In addition, AF can also be divided into sporadic and hereditary cases, with more than 90% of AF cases being sporadic and only 5–10% of cases being hereditary [5, 6]. Sporadic cases predominantly occur in young individuals, particularly women, and are often related to trauma, surgical procedures, or estrogen levels/treatments, while hereditary cases are associated with mutations in familial adenomatous polyposis (FAP) or adenomatous polyposis coli (APC) [7, 8]. It is estimated that about 5–10% of AF cases occur in FAP patients, while about 10–20% of AF patients are found to have an associated FAP or APC gene mutation. APC mutation is a biomarker of FAP, and this mutation is observed in 16% of childhood cases [3].

Although there have been some sporadic cases and cases related to familial adenomatous polyposis reported in the previous literature, the emphasis on details such as the specific surgical techniques and perioperative complications was limited. In this study, we retrospectively analyzed the clinical data of 12 cases of intra-abdominal AF and summarized clinical characteristics, imaging manifestations, and pathological features, especially analyzing the specific surgical details and postoperative complications, aiming to help clinicians increase their understanding of the disease and improve the level of diagnosis and treatment.

## Materials and methods

A total of 12 patients diagnosed with intra-abdominal AF between April 2021 and September 2024 from Xijing hospital, First Affiliated Hospital of Air Force Medical University were included in this study. Inclusion criteria encompassed: (1) Histologically confirmed primary intra-abdominal AF; (2) Surgical resection at our center; (3) Complete clinical and follow-up data. Exclusion criteria encompassed: (1) Patients with FAP-associated AF; (2) Patients who received prior treatment elsewhere. Indications for upfront surgical resection were: (1) presence of symptoms (e.g., pain, mass effect); (2) tumor size > 5 cm with potential for future complications based on location; (3) evidence of tumor growth on serial imaging; (4) patient preference after informed discussion of surveillance versus surgery. Collected data included clinical manifestations, laboratory examination, imaging findings, laboratory results, pathological diagnoses, surgical procedures, postoperative complications and prognosis. The follow-up schedule: clinical exam and imaging every 3–6 months for the first two years, then annually. The follow-up deadline was April 20, 2025. This study was a retrospective, single-center, descriptive case series and was approved by the Ethics Review Committee of Xijing hospital. Written informed consent was obtained from each participant.

Data were analyzed using descriptive statistics. Continuous variables were presented as median (with ranges). Categorical variables were presented as frequencies and percentages. Due to the small sample size and descriptive nature of the study, no comparative or inferential statistical tests were performed.

## Results

### Clinical data

Among the 12 cases of intra-abdominal AF, there were six males and six females, aged 16–59 years (average age: 38 years; median age: 33 years). A retroperitoneal mass was incidentally discovered during physical examination. Three patients presented with pain in the groin, lower abdomen and vaginal tingling. Another two patients were due to recurrence of intra-abdominal AF. Regarding past medical history, two patients had undergone tumor resection at other sites, two had previously undergone intra-abdominal AF resection, one had a history of both gastric stromal tumor and intra-abdominal AF resection, and one patient had undergone total colectomy for familial polyposis coli. The maximum diameter of the tumors ranged from 6 to 30 cm (mean 9 cm). Detailed clinical data are shown in Tables 1 and 3.

### Imaging features and laboratory results

**Fig. 1:**
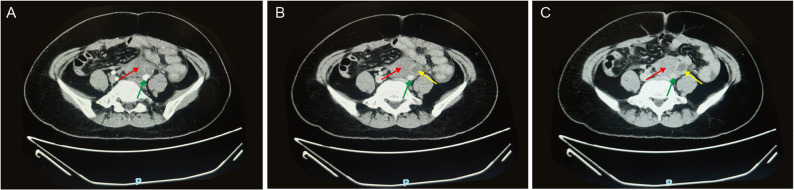
Representative computed tomography images of patients with Intra-abdominal aggressive fibromatosis. Computed tomography revealed a high-density mass (red arrowhead) located in front of the left lumbar psoas muscle, and the left iliac artery (green arrowhead) and left ureter (yellow arrowhead) were completely enveloped by the mass. **A** Enhanced arterial phase. **B** Enhanced venous phase. **C** Enhanced balanced phase

Of the 12 cases with intra-abdominal AF, 10 cases underwent computed tomography (CT) examination, six cases underwent magnetic resonance imaging (MRI) examination, three cases underwent ultrasound examination, and one case underwent PET-CT examination. CT mostly showed irregular soft tissue masses with uneven density and uneven enhancement after enhancement (Fig. 1). MRI showed slightly low signal on T1WI, high signal on T2WI, and significant enhancement on enhanced scan. Ultrasonography showed mixed echo region with unclear boundary and irregular shape. Serum tumor marker analysis revealed that VEGF was elevated in three cases, with a maximum value of 496pg/ml. CA125 was elevated in two cases, with a maximum value of 173U/ml, while other tumor markers such as CA19-9, CEA and AFP were not elevated (Table 1).

**Table 1:** Clinical data from the 12 patients of intra-abdominal aggressive fibromatosis

Clinical features	number (%)
Age(years)	
<18	1(8.33%)
≥18	11(91.67%)
Sex	
Male	6(50%)
Female	6(50%)
Chief complaint	
Retroperitoneal mass	9(66.67%)
Groin pain	1(8.33%)
Abdominal pain	1(8.33%)
Vaginal tingling	1(8.33%)
Medical history	
No medical history	6(50%)
Intra-abdominal AF resection	3(25%)
Total colectomy resection	1(8.33%)
Other tumors resection	3(25%)
Tumor location	
Mesenteric	4(33.33%)
Retroperitoneum	8(66.67%)
Tumor size (maximum diameter, cm)	
≤10	8(66.67%)
11 ~ 20	3(25%)
21 ~ 30	1(8.33%)
Preoperative VEGF (pg/ml)	
≤160	9(75%)
>160	3(25%)
Preoperative CA125 (U/ml)	
≤35	10(83.33%)
>35	2(16.67%)

### Gross and morphological characteristics of specimens

**Fig. 2:**
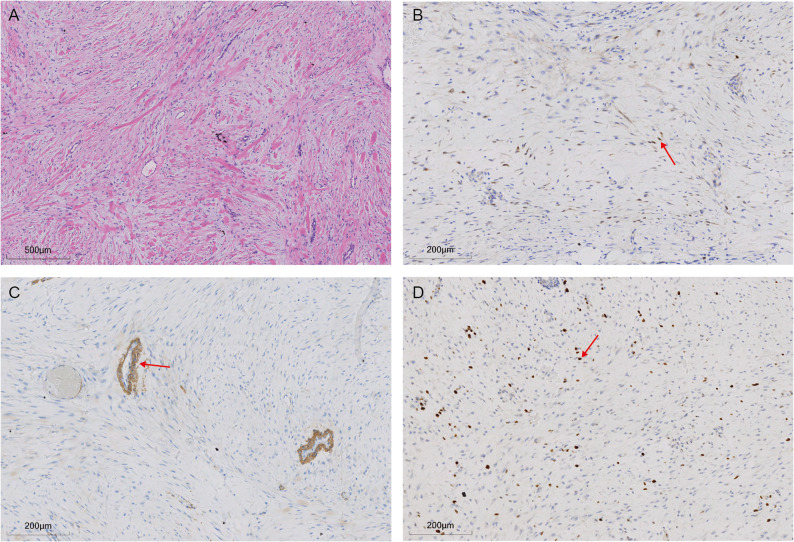
Representative H&E staining (**A**) and immunohistochemical staining (**B**, **C**, **D**) of tumor tissue in Intra-abdominal aggressive fibromatosis. **A** The tumor cells are spindle-shaped, arranged in bundles, whorls or interlacing reticular bundles, and a large amount of collagen deposition can be observed in the interstitium (×4). **B** Nuclear positive for β-catenin protein in tumor cells (red arrowhead, ×10). **C** Positive expression of SMA protein in tumor cells (red arrowhead, ×10). **D** Positive expression of Ki-67 protein in tumor cells (red arrowhead, ×10)

All the tumor specimens were gray, grayish yellow or dark red, and one of them showed gray red nodules with tough texture and no obvious envelope. The section was gray-white, tough and braided, mostly indistinguishable from the surrounding fibrous adipocytes. Microscopically, it consisted mainly of fibroblasts and fibrocytes, collagen fibers interspersed among them. The tumor cells exhibited spindle-shaped, fascicular, mat-like striate, or reticular bundle arrangements. The cellular morphology was benign, with inconspicuous nucleoli and rare nuclear mitosis. Interstitial vessels were abundant with focal bleeding and local mucoid degeneration. A large amount of collagen deposition was seen in the interstitium, and lymphocyte infiltration was seen around it (Fig. 2A).

### Immunohistochemical results of specimens

Tumor cells were positive for β-catenin in 12 cases, SMA in seven cases, Vimentin in four cases, and Ki-67 proliferation index was 2%-20% (Fig. 2B, C, D). Desmin (4/11), S-100 (2/12), SOX10 (1/6), STAT6 (1/7), CD34 (1/12) were positive in a subset of cases. Notably, CD117 was absent in all cases. Additionally, molecular pathologic tests were conducted in three of the 12 cases, and CTNNB1 gene mutations were detected in two of them (Table 2).

**Table 2:** The immunophenotypic results of tumor specimens from 12 patients with intra-abdominal aggressive fibromatosis

patients	β-catenin	Ki-67	Vimentin	SMA	CD34	S-100	CD117	Desmin	STAT6	SOX10	CTNNB1 mutation
1	Nucleus+	2%	་	-	-	-	-				
2	Nucleus+	< 2%	-	-	-	-	-	་	-		
3	Nucleus+	2%	་	་	-	་	-	-	-		-
4	Nucleus+	4%		་	-	-	-	-	-	-	
5	Nucleus+	10%		-	-	-	-	-	་	-	
6	Cytoplasm+	20%		་	-	-	-	་		-	
7	Nucleus+	5%	་	་	་	་	-	་		་	
8	Nucleus+	2%		-	-	-	-	-	-		
9	Nucleus/Cytoplasm+	3–5%		-	-	-	-	-	-	-	
10	Nucleus+	5%		་	-	-	-	་			
11	Nucleus+	5%		་	-	-	-	-	-		་
12	Nucleus+	5%	་	་	-	-	-	-		-	་

Blank cells represent that no detection has been tested;“+”: positive; “-“: negative

### Management strategies and prognosis

Among the 12 cases, the tumors of eight cases were located in the retroperitoneum, while those of four cases were situated in the mesenteric region. The specific tumor locations are shown in Table 3. Ten cases had involvement of other organs or vascular tissues. Among them, six cases had intestinal involvement, one had diaphragmatic involvement, one had left kidney, pancreas and spleen involvement, one had left ureter involvement, and one had right external iliac vein involvement. Accordingly, 10 cases underwent combined organ resection. Four patients developed mild short-term complications such as lower extremity venous thrombosis, ascites, abdominal abscess, anemia and hypoproteinemia after operation. All of them completely recovered after treatment during their hospital stay. All patients did not receive any treatment before the operation and underwent R0 surgical resection without any other adjuvant treatment. The patients were followed up for 7–48 months, with a median follow-up period of 23 months. No recurrence or metastasis occurred during this period.

**Table 3:** Past medical history and history of present illness of 12 patients with intra-abdominal aggressive fibromatosis

patients	Past Medical History			History of Present Illness				
	Surgical time	Clinical diagnosis	Surgical method	Surgical time	Tumor location	Involvement of organ and/or vessel invasion	Surgical method (Apart from tumor resection)	Postoperative complication
1	-	-	-	2022/3/19	the left retroperitoneum and lower thoracic cavity	Diaphragm	Partial diaphragm resection	Lower extremity venous thrombosis
2	2019/10	GIST+ intra-abdominal AF	GIST resection+ intra-abdominal AF resection	2022/6/27	hepatorenal space	Duodenum	Pancreaticoduodenectomy	Lower extremity venous thrombosis+anemia+Hypoproteinemia
3	2022/4	intra-abdominal AF	intra-abdominal AF resection	2022/7/5	left side of the abdominal aorta	Left kidney+Tail of pancreas+Spleen	left kidney +Tail resection of the pancreas+Spleen resection	Abdominal abscess
4	-	-	-	2022/8/18	left side of the abdominal aorta	-	-	-
5	2019/1	Breast cancer	modified radical mastectomy	2022/6/30	left side of the abdominal aorta	Right hemicolon	Right hemicolectomy	-
6	2020/3	Familial colon polyp disease	Total colectomy	2022/11/8	Next to the left iliac artery	Left ureter	Partial resection of the left ureter	-
7	-	-	-	2024/3/20	mesenteric root	Partial small intestine	Partial small intestine resection	Abdominal effusion+Hypoproteinemia
8	2021/4	intra-abdominal AF	intra-abdominal AF resection	2023/4/29	mesenteric root	Partial small intestine	Partial small intestine resection	-
9	-	-	-	2021/4/18	mesenteric root	Part of the colon+Part of the jejunum	Partial colectomy+Partial ileal resection	-
10	2019/7	gastric antrum cancer	Distal gastrectomy	2021/11/18	mesenteric root	Partial small intestine	Partial small intestine resection	-
11	-	-	-	2024/9/14	pelvic retroperitoneum	Right external iliac vein	Right external iliac vein artificial vascular replacement	-
12	-	-	-	2024/8/28	pelvic retroperitoneum	-	-	-

The horizontal line indicates that there is no relevant information; intra-abdominal *AF* intra-abdominal aggressive fibromatosis, *GIST* Gastrointestinal stromal tumors

## Discussion

Aggressive fibromatosis (AF) is a rare disease of deep tissue monoclonal fibroblast proliferation, characterized by no distant metastasis, but with a tendency to infiltrative growth and local recurrence. It exhibits a low incidence rate, accounting for less than 3% of all soft tissue tumors and less than 0.03% of all tumors [2]. AF can occur anywhere in the body, but mainly in the extremities, abdominal wall, head and neck, and the root of the mesentery. The common sites of intra-abdominal AF are mesentery, retroperitoneum or pelvic cavity, while pancreatic and colorectal fibromatosis are very rare. In this study, a total of 12 patients were included, six females and six males. Eight cases were retroperitoneal and four cases were in the mesentery, all of which were single lesions. Intra-abdominal AF is very common in FAP-associated cases, but very rare in sporadic cases, accounting for about 5%-10% of cases [6]. One of the 12 cases occurred two years after surgical resection of familial polyposis coli. Therefore, colonoscopy is necessary for newly diagnosed intra-abdominal AF to rule out the possibility of familial polyposis coli.

The etiology and pathogenesis of AF are not clear. The risk factors of AF are mainly divided into two aspects: non-genetic factors and genetic factors. Non-genetic factors include trauma, surgical history and hormone levels, which are mainly associated with sporadic AF [8]. It is reported that about 30% of AF patients have a history of trauma or surgery, which indicates that the proliferation of fibroblasts may be induced during wound healing and lead to the development of the disease. In this study, six patients (50%) had a history of abdominal surgery. Hormone levels mainly refer to high estrogen status, including estrogen therapy or oral contraceptives, which may explain the slightly higher incidence in women of reproductive age [9]. In addition, genetic abnormalities and syndromes are also major risk factors for AF, especially mutations in familial adenomatous polyposis genes, such as germline APC gene mutations or Gardner syndrome. Most sporadic AF is associated with somatic mutations of the CTNNB1 gene. Mutations in either of these genes lead to alterations in the Wnt/β-catenin pathway, which ultimately leads to uncontrolled proliferation of fibroblasts and promotes AF [10]. However, the specific pathogenesis of AF still needs further study.

Most intra-abdominal AF patients are usually asymptomatic and present with a painless, smooth mass until tumor growth or nearby invasion causes vascular, ureteral, intestinal, or neurological symptoms. Among them, pain is the most prevalent chief complaint and may manifest in areas such as the abdomen and pelvis [11]. Other symptoms include weight loss, fever, nausea, and vomiting [4, 12]. Among the 12 cases reported in this study, nine cases were asymptomatic, while three cases were admitted due to pain (one case with groin pain, one case with lower abdominal pain, and one case with vaginal tingling). In addition, due to the locally aggressive nature, it has been reported that some patients initially present with complications from intra-abdominal AF, including small-bowel obstruction, hydronephrosis, ureteral obstruction or rupture, bowel perforation, enterocutaneous fistula, and intestinal bleeding. Because of its insidious development and nonspecific clinical signs, intra-abdominal AF may be clinically misdiagnosed as an ovarian, mesenteric, or other retroperitoneal tumor [13, 14]. In such cases, meticulous physical examination combined with comprehensive imaging studies must be conducted to prevent diagnostic inaccuracies and inappropriate clinical management.

Imaging examination is essential for the diagnosis of intra-abdominal AF. Imaging modalities encompass ultrasonography, CT, MRI and PET-CT [15–18]. Ultrasonography is a rapid and non-invasive diagnostic method. intra-abdominal AF usually presents as a smooth soft tissue mass with mixed echogenic zones, poorly defined borders, and irregular morphology due to the intermingling of cellular, mucinous, and fibrous components in the tumor tissue [15]. Given its lack of specificity, ultrasonography is usually performed only as a preliminary examination. CT examination is the most commonly used examination method for the diagnosis of intra-abdominal AF and it usually presents as an irregular soft tissue mass. Since the tissue components of the mass include mucous and fibrous connective tissue, the attenuation of the mass can be low density or high density, and tumors show uneven enhancement after enhancement [16, 17]. MRI examination is the imaging method of choice for preoperative assessment and postoperative detection of recurrence because of higher soft tissue resolution and accurate description of the relationship between tumor and surrounding structures. On T1-weighted images, intra-abdominal AF mainly appears as low or equal signal intensity. The signal intensity of T2-weighted image depends on the growth, histological characteristics and biological behavior of the tumor. In the early stage, the tumor tissue components are mainly mucus and cell components, which show high signal intensity on T2-weighted image. With the growth of the tumor and the progress of the disease, fibrous connective tissue proliferation and the relative increase of fibrous components in the tumor components lead to low signal intensity on T2-weighted image [17]. CT and MRI findings were basically consistent with those reported in this study. CT plain scan showed irregular soft tissue mass shadow with uneven density and uneven enhancement after contrast enhancement. MRI plain scan showed slightly low signal intensity on T1-weighted, high signal intensity on T2-weighted, and significant enhancement on enhanced scan. In addition, tumor size and decrease in SUV value monitored by FDG-PET/CT can predict the response to systemic therapy, and volume-based 18F-FDG-PET can also predict the prognosis of intra-abdominal AF [18].

However, the CT and MRI imaging manifestations of AF are not specific and can easily be confused with other tumors such as gastrointestinal stromal tumors (GIST) [14]. Therefore, the diagnosis mainly relies on clinical pathological examination. Pathological specimens can be obtained by ultrasound-guided core needle biopsy or surgical exploration. Histologically, AF is characterized by the presence of uniformly shaped spindle-shaped cells within an abundant collagen matrix and vascular network, and these cells exhibit a monoclonal proliferation pattern closely resembling that of myofibroblasts [19]. In this study, all the pathological specimens were obtained by surgical resection. IHC results showed that β-catenin was positive in all specimens. Ki-67 index ranged from 2% to 20%. SMA, Vimentin, Desmin, S-100, SOX10, STAT6 and CD34 were positive in some cases, while CD117 was negative in all cases. A study showed that 80% of intra-abdominal AF is positive for nuclear expression of β-catenin, and cases with FAP are more common [20]. N.Ogawa et al. reported that positive nuclear β-catenin and negative CD34 are the unique characteristics of intra-abdominal AF and are often used to distinguish intra-abdominal AF from GIST [21]. IHC showed nuclear β-catenin positivity in all 12 cases, indicating that this marker has high sensitivity and specificity for the diagnosis of intra-abdominal AF, a finding potentially linked to the Wnt/β-catenin pathway [22]. One of the 12 cases was positive for CD34, indicating that CD34 expression is not specific for the differential diagnosis of intra-abdominal AF and GIST. Therefore, the differential diagnosis of AF from other soft tissue tumors mainly relies on the combination of IHC markers rather than a single marker. Among them, positive nuclear β-catenin is the core feature of AF. The IHC results such as CD117 and CD34 are used as auxiliary markers for the diagnosis of AF. Most cases of AF are CD117-negative, and almost all are CD34-negative.

According to the global consensus guidelines established by the Global Fibromatosis Working Group in 2019, the treatment of AF involves systemic treatment based on active monitoring, combined with local treatment methods such as surgery, radiotherapy and medication [23–28]. For severe symptoms, rapid progression or serious complications, surgical treatment is still the first-line treatment. Especially for abdominal wall and intra-abdominal AF, surgery remains the main treatment after disease progression [23]. Furthermore, surgical treatment is preferable for patients who are unlikely to comply with active surveillance. Traditionally, the primary principle of surgery is complete resection of the tumor and negative surgical margins to reduce the recurrence rate of the tumor. However, different studies have suggested that the value of surgical margin status of intra-abdominal AF is controversial. It has been reported that one case that underwent partial resection had recurrence and the other 10 cases who underwent R0 resection had no recurrence among 11 cases with intra-abdominal AF who underwent surgical resection (one case was non-R0 resection, ten cases were R0 resection), suggesting that the status of surgical margin is related to the recurrence rate [20]. In contrast, some studies have shown that the postoperative recurrence rate is not related to surgical margin. A retrospective study demonstrated that R1 resection margin status was not associated with an increased risk of local recurrence or compromised 5-year progression-free survival compared to R0 resections [29]. Another retrospective analysis also confirmed that microscopic positive resection margin does not have any effect on the prognosis of patients with intra-abdominal AF [30]. The findings of this study provide important clinical evidence for this controversy. Among 12 patients, the tumors were located in the mesentery of the small intestine (four cases) and the retroperitoneum (eight cases), with an average maximum diameter of 9 cm. It is notable that 10 patients (83.3%) underwent combined organ resection due to tumor invasion of surrounding organs and blood vessels, including intestinal resection, spleen resection, kidney resection, or partial pancreatic resection. Nevertheless, all cases were confirmed to have undergone R0 resection (negative margins) by postoperative pathology. The 100% R0 resection rate fully demonstrates the experience and technical advantages of our center in complex abdominal surgeries, and also reflects the biological characteristic of intra-abdominal AF that is locally invasive. Among 12 patients, four cases (33.3%) experienced short-term complications after the operation, including lower extremity venous thrombosis, abdominal effusion, abdominal abscess, anemia, and hypoproteinemia. Patients recovered from these complications during the hospital stay through conservative treatment, and no serious adverse events such as anastomotic leakage, secondary surgery, or death occurred. This indicates that even if intra-abdominal AF requires combined organ resection, with the implementation of early surgery by an experienced surgical team, the complications can still be controlled within an acceptable range. In terms of long-term efficacy, the results of this cohort are encouraging. During the median follow-up of 23 months (range 7 to 48 months), all 12 patients did not experience recurrence or metastasis. Even if combined organ resection is required, patients can still achieve good short-term recurrence-free survival under the condition of achieving R0 resection.

One limitation of this study is that it is a single-center retrospective study with a relatively small sample size and no control group. This limits the power of the statistical analysis and may lead to selection bias in the results. Therefore, these findings should be interpreted as descriptive and hypothesis-generating rather than definitive. Further large-scale prospective studies are still needed to determine the association between the surgical margins of intra-abdominal AF surgery and local recurrence. Secondly, the follow-up period is relatively short. Although no recurrence was observed in the short term, AF mostly has late recurrence. Therefore, the long-term efficacy still needs to be continuously verified through follow-up. Currently, we are conducting regular follow-ups and tracking for these patients, and the follow-up data will be updated subsequently. Thirdly, only 3 patients (25%) in this group underwent CTNNB1 mutation detection. Therefore, the observed mutation frequency (2/3 positive) cannot represent the entire cohort and cannot be used for the association analysis between CTNNB1 mutations and clinical outcomes. Future studies should conduct systematic molecular testing for all enrolled cases to deeply explore the role of gene mutations in the occurrence, development, and prognosis assessment of intra-abdominal AF.

## Conclusions

In summary, we reported 12 patients with intra-abdominal AF who underwent upfront surgical resection. All achieved R0 resection, with 83.3% requiring combined organ resection, yet postoperative complications were mild (33.3%) and all resolved during hospitalization. At a median follow-up of 23 months, no recurrence occurred. These findings suggest that patients with intra-abdominal AF can achieve complete resection with acceptable morbidity and favorable short-term outcomes in experienced centers. Due to the small sample size, short follow-up, and incomplete CTNNB1 testing, these observations should be considered preliminary and hypothesis-generating rather than definitive, and these findings still require validation through long-term follow-up in multi-center prospective studies.

## Data Availability

The datasets used and/or analysed during the current study are available from the corresponding author on reasonable request.
